# Effect of Ascorbic Acid Addition on the Phenolic Compounds Content in Homogenates from Aerial Parts of Spearmint, Fennel, and Thyme

**DOI:** 10.3390/foods14132165

**Published:** 2025-06-21

**Authors:** Jan Tříska, Naděžda Vrchotová, Jan Strohalm, Milan Houška, Eliška Kováříková, Pavla Novotná, Jan Bednář, Roman Pavela

**Affiliations:** 1 Global Change Research Institute, CAS, Bělidla 986/4a, 603 00 Brno, Czech Republic; vrchotova.n@czechglobe.cz (N.V.); bednar.j@czechglobe.cz (J.B.); 2 Faculty of Agriculture and Technology, University of South Bohemia, Studentská 1668, 370 05 České Budějovice, Czech Republic; 3 Czech Agrifood Research Centre, Drnovská 507, Ruzyně, 161 06 Prague, Czech Republic; jan.strohalm@carc.cz (J.S.); milan.houska@carc.cz (M.H.); eliska.kovarikova@carc.cz (E.K.); pavla.novotna@carc.cz (P.N.); roman.pavela@carc.cz (R.P.); 4 Department of Plant Biotechnology, College of Life Sciences and Biotechnology, Korea University, Seoul 02841, Republic of Korea

**Keywords:** herbs homogenates, *Mentha spicata*, *Foeniculum vulgare*, *Thymus vulgaris*, phenolic compounds, ascorbic acid addition

## Abstract

The paper deals with the investigation of the ascorbic acid influence on the analytical results of polyphenol content in the samples of the spearmint, fennel, and thyme homogenates. The homogenates without and with addition of ascorbic acid and water were prepared and stabilized by high-pressure treatment. Their analysis was accomplished by high-performance liquid chromatography (HPLC) with DAD detection and by combination of liquid chromatography with mass spectrometry (LC-MS). Volatile terpenes were analyzed in all homogenates by combination of gas chromatography with mass spectrometry technique (GC-MS). The content of polyphenols of acidic nature, e.g., rosmarinic acid, revealed the highest difference between analytical results of the samples with and without ascorbic acid. Finally, prepared herb homogenates are suitable food supplements, which will find increasing application in various food preparations.

## 1. Introduction

Spearmint (*Mentha spicata*), fennel (*Foeniculum vulgare*), and thyme (*Thymus vulgaris*) are the most common herbs used not only in folk medicine, in the kitchen, and in the food industry, but the attention is increasingly being paid to them in modern pharmacy and in bio industry (e.g., products with insecticidal activity). The mentioned plants contain a number of phenolic biologically active substances e.g., diosmin, diosmetin, hesperidin, luteolin, apigenin, rosmarinic acid in spearmint [1,2,3], chlorogenic acid, miquelianin, 1,5-dicaffeoylquinic acid, kaempferol-3-glucuronide, kaempferol-3-arabinoside in fennel [4,5] and luteolin-7-glucuronide, apigenin-7-glucuronide, rosmarinic acid, and salvianolic acid derivatives in thyme [6,7]. The dominant terpenic substances include e.g., carvacrol, thymol, p-cymene in thyme [8], γ-terpinene, 4-allylanisole, anethole, limonene in fennel [9,10,11] and limonene, 1,8-cineole, carvone, and cymene in spearmint [12,13,14,15].

An overview of phenolic substances and their testing in pharmacy is provided by several reviews, e.g., on the effects of spearmint substances [16,17,18], thyme substances [18,19], and fennel substances [20,21].

High-pressure processing (HPP) is technology used for food processing and is denoted cold pasteurization. Products in suitable packaging are subjected to isostatic pressure 300–600 MPa. This process inactivates the most active forms of microorganisms and preserves the sensorial and nutritional quality of original raw materials [22].

Regarding the fact that the herb homogenates with a guaranteed content of active substances find increasing application in various preparations and food supplements, e.g., sage homogenate [23], it means that the homogenates must be standardized in some way. The technology of stabilizing homogenates has not yet been sufficiently studied, especially regarding the content of health-promoting substances. One way to preserve the content of biologically active substances could be acidification. Acidification is an effective process to minimize the risk of bacterial spore germination and subsequently to spoil fruit and vegetable juices treated with high pressure [24]. The goal of our work was, therefore, to determine the effect of the addition of ascorbic acid on the terpenic and phenolic substances content in the final spearmint, fennel, and thyme homogenates.

## 2. Materials and Methods

### 2.1. Plant Materials

*Mentha spicata* (spearmint), *Foeniculum vulgare* (fennel), and *Thymus vulgaris* (thyme) were grown on the grounds of the Research Institute of Plant Production in Prague and Olomouc.

### 2.2. Preparations of Homogenates

The aerial parts of fresh herb material were homogenized with or without the additions of water and ascorbic acid in different ratio, and the final pH value of the mixture was measured. The experimental setup and pH value are given in Table 1.

Fresh herbs were collected, and a small part was spread on sieves and allowed to dry for determination of dry matter before being placed in a cold room. Subsequently, homogenization, i.e., mechanical disintegration of aerial parts of herbs, was performed using mixer Coupe R301 (Montceau-en-Bourgone, France) with individual amounts of water because plants differ in dry matter and toughness of aerial parts. The method is based on mechanical disintegration and must be adapted with respect to the individual state of plant tissue. Similarly, ascorbic acid dose was adapted with pH level and is individual for each plant. The dose was evaluated with respect to pH lowering to level 3.8–4.5. The procedure for homogenizing fresh herbs into a smooth paste that is useful for food preparation was developed in our laboratory and has not been previously published in international journals.

### 2.3. Microbial Analysis

A horizontal method for the enumeration of microorganisms by colony count at 30 °C by ISO 4833-1 [25] was used. ISO 21527-1:2008 [26] was followed for the number of yeast and molds determination. Plate count agar (Himedia, Maharashtra, India) was used for total count of microorganisms, and Yeast Glucose Chloramphenicol (YGC) Agar (Sigma-Aldrich, Prague, Czech Republic) was used for yeast and mold cultivation. Samples were cultivated at 30 °C, and colony-forming units (CFU) were enumerated.

### 2.4. High-Pressure Processing

Samples given in Table 1 represent homogenates that were filled into polyethylene terephthalate/aluminum/polyethylene (PET/Al/PE) containers, which were vacuum sealed and then treated with a high pressure of 500 MPa for 10 min, then cooled to 15 °C and stored in refrigerator between 5 and 8 °C. The pressurizing was performed by a high-pressure press CYX 6/0103 (ŽĎAS a. S., Žďár nad Sázavou, Czech Republic). High-pressure treatment was used as an antimicrobial intervention replacing pasteurization. Pressurized homogenates are microbially stabile for at least 2 years for food applications.

### 2.5. Extraction of Phenolic Compounds from Homogenate

The phenolic compounds were extracted from homogenates by methanol similarly as in [7], but at the higher temperature and for a shorter time. Then, 0.25 g of homogenate were extracted by 3 mL of 100% methanol, and the extraction was conducted at 50 °C for 1 h. After centrifugation (3500 rpm, 10 min), the sediment was washed twice with 1 mL of methanol. Supernatants were combined and the total volume was measured. Each sample was prepared in triplicate and stored at −18 °C. The extracts were analysed using HPLC and LC/MS.

### 2.6. Extraction of Terpenes from Homogenates

Samples of plant material homogenates (thyme, fennel, spearmint) were prepared identically in the following manner. Approximately 0.3 g of homogenate were weighed in triplicates from each sample (double the amount for water-diluted homogenates). The weighed amount was extracted three times with 2 mL of hexane. After the first addition of hexane, the mixture was shaken for 1 h. The hexane was then collected in a separate vial. Then, 2 mL of hexane were added again to the homogenate. This mixture was shaken for 0.5 h and then hexane was added to the first portion of hexane. Finally, 2 mL of hexane were added again and shaken for 0.5 h. The last portion of hexane was also added to the previous two. The hexane solution thus obtained was directly injected into the GC/MS. The hexane extract obtained from spearmint homogenates was diluted five times before measurement due to high concentration of the components.

### 2.7. Determination of Phenolic Compounds

Quantification of phenolic compounds by HPLC: The samples were analysed using an HPLC apparatus (Hewlett Packard 1050) (Hewlett-Packard, Palo Alto, CA, USA) with a diode array detector (DAD Agilent G1315B, Palo Alto, CA, USA) and column Phenomenex Luna C18(2) (3 µm, 2 × 150 mm) (Phenomenex, Torrance, CA, USA).

Mobile phase A: 5% acetonitrile + 0.1% *o*-phosphoric acid, mobile phase B: 80% acetonitrile + 0.1% *o*-phosphoric acid. Gradient for separation of fennel (35 °C): 9% to 33% of B during 35 min, 33% to 45% of B during 1 min, 45% to 80% of B during 2 min, 80% to 100% of B during 2 min. Gradient for separation of spearmint (25 °C): 2% to 42% of B during 40 min, 40% to 80% of B during 2 min, 80% of B during 3 min. Gradient for thyme (35 °C): 0% to 45% of B during 55 min, 45% to 80% of B during 5 min. The volume of the injected sample was 5 µL. Flow rate was 0.25 mL/min.

Standards (diosmin, diosmetin, hesperidin, rosmarinic acid, luteolin, apigenin, chlorogenic acid, miquelianin, 1,5-dicaffeoylquinic acid, luteolin-7-glucuronide) were purchased from Sigma-Aldrich, Praha, Czech Republic; methanol and acetonitrile from Merck, Praha, Czech Republic, *o*-phosphoric acid from Fluka, formic acid from Sigma-Aldrich, Praha, Czech Republic.

Identification of phenolic compounds was performed by LC/MS. For compound identification, we used atmospheric pressure chemical ionization (APCI-LC/MS) in positive and negative mode. The instrument (LCQ Accela Fleet, Thermo Fisher Scientific, San Jose, CA, USA) had the same column and used the same gradients as in HPLC, but mobile phases were acidified by 0.1% formic acid. Instrument conditions were the following: Vaporizer temperature 300 °C, sheath gas flow rate 58 L/min, auxiliary gas flow rate 10 L/min, discharge current 5 µA, capillary temperature 275 °C, and capillary voltage 2 V.

### 2.8. Determination of Volatile Terpenes

Terpenes from homogenates extracts were analysed on a Trace GC Ultra gas chromatograph (Thermo Fischer Scientific, San Jose, CA, USA) equipped with a Restek-fused silica capillary column, Rxi-5 ms, 30 m × 0.25 mm I.D. × 0.25 µm (Restek Corporation, Bellefonte, PA, USA), liner SKY, Splitless, 3 mm × 0.8 mm × 105 mm (Restek Corporation), and coupled to a mass selective detector ISQ (Thermo Fischer Scientific) working at 70 eV of ionization energy. Helium was used as a carrier gas at 1.0 mL/min with injection of 1 µL in splitless mode at 250 °C. Split flow after 1 min was 50 mL/min. The oven temperature was programmed as follows: 40 °C for 5 min, then increase to 150 °C at a rate of 3 °C/min, further increase to 250 °C at 10 °C/min, and finally increase to 290 °C at a rate of 25 °C/min. This temperature was then held for 2 min. Transfer line temperature was 250 °C, ion source temperature was set to 200 °C. Mass scanning was started at 7.00 min, masses were scanned in the full range 50–450 m/z. Qualitative analysis was performed using 36 purchased standards, and their list is given in the table in the Supplementary Materials.

### 2.9. Statistics

A Two-Way ANOVA [27] was conducted to determine to what extent H_2_O and ascorbic acid have an influence on contents of specific compounds.

## 3. Results and Discussion

Plant homogenates were prepared as shown in Materials and Methods in Table 1.

Microbial stability of homogenates with water and ascorbic acid was suitable for food application during 21 days of storage (see Table 2). The number of microorganisms did not exceed 1.3 × 10^3^ during storage in laboratory temperature. Yeast and mold were not detected in all samples during storage period. Microbial stability of homogenates with ascorbic acid can be supported by pH lower than 4.5 and antimicrobial impact of phytochemical components.

The results of analyses of the phenolic compounds of interest (Table 3) showed that the differences in the content of most substances in different homogenate preparation were considerable. The greatest effect has the addition of ascorbic acid during the homogenization process on the measured content of phenolic acids, e.g., chlorogenic acid, rosmarinic acid, and their derivatives, while the measured number of phenolic acids is minimal without addition of ascorbic acid. In thyme homogenates with ascorbic acid addition, the measured rosmarinic acid content is 37,212 mg/kg d. m., while in thyme homogenates without ascorbic acid, the measured content of rosmarinic acid is only 196 mg/kg d. m., and in thyme homogenates without ascorbic acid but with water addition, its content is even a little less (101 mg/kg d. m.) (Table 3). It is obvious that measured content of phenolic acids is higher in the presence of ascorbic acid and results in lower pH value. Better stability of chlorogenic acid at low pH (pH 3) was described already in the literature [28], but extraction is influenced also by water addition, and statistically significant effect has water and ascorbic acid simultaneously. The higher content was also observed by the following flavonol glucuronides (quercetin-3-O-glucuronide (miquelianin), other quercetin derivative and kaempferol-3-O-glucuronide) and flavone glucuronides (luteolin-7-glucuronide and apigenin-7-glucuronide). All mentioned glucuronides have the highest content in the homogenates with added ascorbic acid whether water is added or not. This is due to the greater stability of glucuronides at lower pH [29]. Extraction of mentioned flavonols is also influenced by water addition, ascorbic acid presence, and their combination. Only quercetin derivatives in *Foeniculum vulgare* do not show a statistically significant difference for the combined use of water and ascorbic acid. Kaempferol-arabinoside also shows greater availability in the presence of ascorbic acid and water.

Regarding flavan hesperidine, its content is highest in homogenates with ascorbic acid addition but without the addition of water. Combination of other conditions also influences the yield of extraction. The other flavones (luteolin, diosmin, diosmetin) are influenced with water and ascorbic acid presence in homogenate, and with their combination. Apigenin behaves rather differently. There is an unclear dependence on the presence of ascorbic acid, but from a statistical point of view, only water addition has a significant effect (Table 3). In the opposite, the highest content of diosmetin was found in all homogenates without ascorbic acid addition. The content of diosmin is the highest in homogenate with water addition, but without ascorbic acid addition; in water-free homogenates with or without ascorbic acid addition, the content of diosmin is lower. In this context, it is clear that herb homogenate preparation and their analysis are very important steps, as our results show. The greatest changes are in the rosmarinic acid content. Pure rosmarinic acid is a stable substance, and its solution in ethanol is also stable at different temperatures (10–40 °C) and under different light exposure, as experimentally found [30].

Rosmarinic acid content in *Melissa officinalis* tinctures prepared from dry plant material was higher (2.96–22.18 mg/mL) than in the tinctures prepared from fresh ground-crushed material (less than 0.92 mg/mL) [31]. Olah et al. [32] found higher rosmarinic acid content in fresh *Rosmarinus officinalis* tinctures (0.35 mg/mL) than in the tinctures prepared from dried material (0.18 mg/mL), but it should be noted that the above-mentioned authors did not cut or crush fresh material. Thus, the enzymatic activity was not increasing. Six et al. [33] found that the amount of rosmarinic acid in a 50% ethanolic extract from the dried material of various Laminaceae decreases after 24 weeks by 14–27%, even by 41% in sage. Bodalska et al. [34] tested the stability of rosmarinic acid in commercial herbal medicinal products and found that in aqueous tinctures, rosmarinic acid is very unstable; stability is much better in water-ethanolic extracts. A very important step influencing the final content of the compounds of acidic nature is whether we will grind the samples or leave the whole fresh plant material intact [35]. In the fresh plant homogenates, the released enzymes act on chlorogenic and rosmarinic acids and their derivatives, and these compounds are, therefore, the subject of rapid degradation. The presence of ascorbic acid has at least dual positive effects on the content of the compounds. First, ascorbic acid decreases pH and low pH blocks enzymatic activity; and second, ascorbic acid suppresses ionization of acidic compounds in water. Therefore, they reveal higher yield during the extraction step. The content of rosmarinic acid in homogenates from *Mentha spicata* and *Thymus vulgaris* was influenced not only by ascorbic acid addition, but also with water addition. The combination of these two additions has also a statistically significant effect. In both homogenates, without ascorbic acid addition, rosmarinic acid content was very low. The same was observed also for the rosmarinic acid derivatives 1 and 2. For extraction of rosmarinic acid and their derivatives, the presence of water is also important; only rosmarinic acid derivative 1 was not influenced by water addition.

In acidic media, terpenes generally undergo various transformations. The complex mixtures of products obtained in these transformations are the major factors that hinder the proper interpretation of the analytical results, especially because essential oils (EOs) themselves are a very complex matrix. In addition, these processes can also be influenced by the phenolic substances present, as is the case, for example, with γ-terpinene. In the case of γ-terpinene, we can see the largest changes in the homogenate from *Thymus vulgaris* in Table 4. In the presence of ascorbic acid and water, the amount of γ-terpinene increases from 0.377 to 1.510 (measured by the relative peak area—here and further in the discussion). The opposite is true for 4-cymene. The content decreased in this homogenate from 3.3 to 0.593, as well as in *Foeniculum vulgare* homogenate from 0.570 to 0.110. In this homogenate, the largest decrease in α-pinene was also observed, from 0.717 to 0.257. In contrast, this decrease in β-pinene content was not observed in the acidic environment in the *Mentha spicata* homogenate.

It is clear from the literature [22] that for most plant enzymes, the response to pressure-induced inactivation is enzyme-specific and depends on the conditions applied with partial inactivation at most under commercially feasible HPP conditions. In general, enzymes are more resistant to inactivation than vegetative microorganisms, posing a challenge to the application of HPP for stabilization of fruit and vegetable products.

By using herb homogenates as a food additive, a complex of substances having the same synergistic effect and the same biological effects as the original herb is introduced into the food, which is not ensured by adding only one major substance obtained from the herb by extraction. This was verified in the hops homogenate, where the complete homogenate had higher antimicrobial activity than individual alpha and beta bitter acids acting alone [36]. It is quite clear that plant extracts and herb homogenates in this case have shown a considerable promise in a range of applications in the food industry, which also results from the published literature, e.g., [37].

## 4. Conclusions

The differences in the content of most polyphenolic compounds in different homogenate preparation were considerable. The greatest effect has the addition of ascorbic acid during the homogenization process on the content of phenolic acids, e.g., rosmarinic acid and chlorogenic acid. In thyme homogenates, the rosmarinic acid content was almost 200 times higher compared to thyme homogenates without ascorbic acid. The content of flavonols glucuronides (quercetin-3-O-glucuronide, kaempferol-3-O-glucuronide) and flavone glucuronides (luteolin-7-glucuronide and apigenin-7-glucuronide) was also the highest in the presence of ascorbic acid. Flavones luteolin, apigenin, diosmin, and diosmetin behave rather differently; there is an unclear dependence on the presence of ascorbic acid. From our experiments, and from the literature, we can conclude that for the preservation of phenolic acids, flavonols, and flavone glucuronides in the fresh herb homogenates, it is very important to add ascorbic acid, which will block enzymatic activity. This step prevents rapid degradation of the compounds and suppresses ionization of acidic compounds in water. Therefore, compounds of acidic nature reveal a higher amount in the homogenates. The obtained results also show that in order to achieve the necessary accuracy in the analysis of natural substances containing phenolic compounds and terpenes, increased attention must be paid to setting and maintaining the optimal pH value.

## Figures and Tables

**Table 1:** Compositions and pH of the homogenates.

Sample	Sample Composition	pH Value
M-1	400 g M	6.17
M-2	400 g M + 400 g W	6.60
M-3	400 g M + 18 g AA	4.24
M-4	400 g M + 400 g W + 18 g AA	4.34
F-1	400 g F	5.71
F-2	400 g F + 200 g W	5.59
F-3	348 g F + 30 g W + 6 g AA	4.17
F-4	400 g F + 200 g W + 10 g AA	4.18
T-1	400 g T	6.20
T-2	200 g T + 300 g W	6.18
T-3	300 g T + 18 g AA	3.91
T-4	400 g T + 600 g W + 24 g AA	3.91

M…*Mentha spicata*, F…*Foeniculum vulgare*, T…*Thymus vulgaris*, W…water, AA…ascorbic acid.

**Table 2:** The microbial stability of homogenates prepared with water and ascorbic acid.

Sample	Time of Storage (Day)	Total Count (CFU/g) at 5 °C	Total Count (CFU/g) at 20 °C
M-4	0	1.1 × 10^3^	1.1 × 10^3^
	7	1.3 × 10^3^	1.1 × 10^3^
	14	1.1 × 10^3^	1.1 × 10^3^
	21	9.1 × 10^2^	9.3 × 10^2^
F-4	0	8.2 × 10^1^	8.2 × 10^1^
	7	7.7 × 10^2^	8.0 × 10^2^
	14	3.7 × 10^2^	3.3 × 10^2^
	21	5.0 × 10^2^	1.7 × 10^2^
T-4	0	6.1 × 10^1^	6.1 × 10^1^
	7	3.8 × 10^2^	1.4 × 10^2^
	14	2.3 × 10^2^	1.6 × 10^2^
	21	2.5 × 10^2^	1.7 × 10^2^

**Table 3:** The content of monitored phenolic compounds (mg/kg dry matter).

*Mentha spicata*	Water	Ascorbic Acid	Diosmin	Hesperidin	Rosmarinic Acid	Luteolin	Apigenin	Diosmetin
M-1	0	0	12,826 ± 524 ^abc^	893 ± 46 ^abc^	122 ± 27 ^abc^	485 ± 26 ^abc^	485 ± 17 ^a^	922 ± 111 ^abc^
M-2	water	0	22,364 ± 1039 ^abc^	1201 ± 66 ^abc^	95 ± 4 ^abc^	652 ± 29 ^abc^	571 ± 60 ^a^	1888 ± 143 ^abc^
M-3	0	ascorbic acid	13,862 ± 842 ^abc^	2471 ± 144 ^abc^	66,588 ± 2393 ^abc^	495 ± 31 ^abc^	495 ± 46 ^a^	309 ± 44 ^abc^
M-4	water	ascorbic acid	18,573 ± 571 ^abc^	1281 ± 106 ^abc^	33,271 ± 2013 ^abc^	1000 ± 25 ^abc^	599 ± 9 ^a^	720 ± 26 ^abc^
***F*** ***oeniculum vulgare***	**Water**	**Ascorbic Acid**	**Chlorogenic Acid**	**Miquelianin**	**Quercetin Derivative**	**1,5-dicaffeoylquinic Acid**	**Kaempferol-3-** ***O*** **-Glucuronide**	**Kaempferol-3-** ***O*** **-Arabinoside**
F-1	0	0	n.d.	527 ± 38 ^abc^	372 ± 28 ^ab^	n.d.	495 ± 26 ^abc^	443 ± 17 ^abc^
F-2	water	0	n.d.	555 ± 41 ^abc^	309 ± 16 ^ab^	81 ± 18 ^abc^	481 ± 17 ^abc^	420 ± 15 ^abc^
F-3	0	ascorbic acid	1864 ± 46 ^abc^	1933 ± 55 ^abc^	889 ± 23 ^ab^	716 ± 38 ^abc^	798 ± 19 ^abc^	579 ± 17 ^abc^
F-4	water	ascorbic acid	2021 ± 40 ^abc^	2416 ± 66 ^abc^	853 ± 28 ^ab^	617 ± 10 ^abc^	1098 ± 35 ^abc^	664 ± 28 ^abc^
***Thymus vulgaris***	**Water**	**Ascorbic Acid**	**Luteolin-7-Glucuronide**	**Apigenin 7-Glucuronide**	**Rosmarinic Acid**	**Rosmarinic Acid Derivative 1**	**Rosmarinic Acid Derivative 2**	**Caffeoyl-Rosmarinic Acid**
T-1	0	0	563 ± 3 ^abc^	1476 ± 32 ^abc^	101 ± 8 ^abc^	n.d.	n.d.	n.d.
T-2	water	0	954 ± 81 ^abc^	2550 ± 280 ^abc^	196 ± 63 ^abc^	n.d.	n.d.	n.d.
T-3	0	ascorbic acid	7476 ± 39 ^abc^	2608 ± 90 ^abc^	37,212 ± 992 ^abc^	3737 ± 212 ^b^	4098 ± 127 ^abc^	3061 ± 145 ^abc^
T-4	water	ascorbic acid	8734 ± 84 ^abc^	3011 ± 89 ^abc^	33,145 ± 255 ^abc^	3779 ± 174 ^b^	4611 ± 97 ^abc^	2775 ± 44 ^abc^

a—influenced by water addition, b—influenced by ascorbic acid addition, c—influenced by both water and ascorbic acid addition, chlorogenic acid LOD 0.42 µg·mL^−1^; 1,5-dicaffeoylquinic acid LOD 0.55 µg·mL^−1^; rosmarinic acid LOD 0.079 µg·mL^−1^. n.d. not detected.

**Table 4:** The content of monitored terpenes (% of peaks from normalized peak area).

*Mentha spicata*	Water	Ascorbic Acid	β-Pinene	Myrcene	Limonene	Eucalyptol	*trans*-Caryophyllene	Piperitenone Oxide	
M-1	0	0	0.337 ± 0.017 ^ac^	1.090 ± 0.029 ^abc^	2.517 ± 0.168 ^ab^	5.443 ± 0.076 ^b^	1.883 ± 0.125 ^ac^	83.010 ± 0.403 ^ac^	
M-2	water	0	0.350 ± 0.008 ^ac^	1.140 ± 0.041 ^abc^	2.583 ± 0.078 ^ab^	5.563 ± 0.181 ^b^	1.537 ± 0.024 ^ac^	82.500 ± 0.268 ^ac^	
M-3	0	ascorbic acid	0.313 ± 0.005 ^ac^	1.170 ± 0.037 ^abc^	2.623 ± 0.063 ^ab^	6.007 ± 0.046 ^b^	1.800 ± 0.033 ^ac^	83.303 ± 0.172 ^ac^	
M-4	water	ascorbic acid	0.367 ± 0.012 ^ac^	1.457 ± 0.026 ^abc^	2.980 ± 0.128 ^ab^	6.007 ± 0.118 ^b^	1.720 ± 0.028 ^ac^	81.890 ± 0.168 ^ac^	
***Foeniculum vulgare***	**Water**	**Ascorbic Acid**	**α-Pinene**	**Phelandrene**	**4-Cymene**	**Limonene**	**Fenchone**	**Estragole**	***trans-*** **Anethole**
F-1	0	0	0.717 ± 0.005 ^abc^	1.153 ± 0.005 ^ac^	0.570 ± 0.008 ^bc^	0.217 ± 0.005 ^ab^	1.160 ± 0.033 ^ab^	0.260 ± 0.014 ^abc^	95.800 ± 0.059 ^abc^
F-2	water	0	0.480 ± 0.054 ^abc^	0.877 ± 0.052 ^ac^	0.780 ± 0.078 ^bc^	0.173 ± 0.017 ^ab^	1.323 ± 0.068 ^ab^	0.383 ± 0.009 ^abc^	95.863 ± 0.130 ^abc^
F-3	0	ascorbic acid	0.650 ± 0.008 ^abc^	1.295 ± 0.004 ^ac^	0.210 ± 0.008 ^bc^	0.160 ± 0.000 ^ab^	0.775 ± 0.029 ^ab^	0.250 ± 0.024 ^abc^	96.595 ± 0.061 ^abc^
F-4	water	ascorbic acid	0.257 ± 0.012 ^abc^	0.817 ± 0.024 ^ac^	0.110 ± 0.008 ^bc^	0.110 ± 0.008 ^ab^	0.750 ± 0.024 ^ab^	0.223 ± 0.005 ^abc^	97.650 ± 0.029 ^abc^
***Thymus vulgaris***	**Water**	**Ascorbic Acid**	**4-Cymene**	**γ-Terpinene**	**Linalool**	**Borneol**	**Terpinen-4-ol**	**Thymol**	**Carvacrol**
T-1	0	0	3.300 ± 0.062 ^ab^	0.377 ± 0.031 ^abc^	0.177 ± 0.046 ^a^	0.230 ± 0.043 ^a^	0.057 ± 0.017 ^abc^	88.897 ± 0.893 ^ac^	2.760 ± 0.228 ^abc^
T-2	water	0	2.733 ± 0.070 ^ab^	0.257 ± 0.012 ^abc^	0.100 ± 0.000 ^a^	0.120 ± 0.008 ^a^	0.027 ± 0.005 ^abc^	91.180 ± 0.204 ^ac^	2.510 ± 0.070 ^abc^
T-3	0	ascorbic acid	1.147 ± 0.021 ^ab^	2.243 ± 0.108 ^abc^	0.207 ± 0.029 ^a^	0.220 ± 0.033 ^a^	0.313 ± 0.025 ^abc^	86.267 ± 1.319 ^ac^	3.967 ± 0.180 ^abc^
T-4	water	ascorbic acid	0.593 ± 0.056 ^ab^	1.510 ± 0.104 ^abc^	0.090 ± 0.000 ^a^	0.100 ± 0.000 ^a^	0.200 ± 0.000 ^abc^	92.663 ± 0.081 ^ac^	2.750 ± 0.273 ^abc^

a—influenced by water addition; b—influenced by ascorbic acid addition; c—influenced by both water and ascorbic acid addition. "0" in the Table means that there is no addition of ascorbic acid or water.

## Data Availability

The original contributions presented in the study are included in the article, further inquiries can be directed to the corresponding author.

## Supplementary Materials

The following supporting information can be downloaded at: https://www.mdpi.com/article/10.3390/foods14132165/s1.
