# Supplementary material for: Effect of Ascorbic Acid Addition on the Phenolic Compounds Content in Homogenates from Aerial Parts of Spearmint, Fennel, and Thyme
Source: Foods. 2025 Jun 21;14(13):2165. doi: 10.3390/foods14132165 (PMC12248822; doi:10.3390/foods14132165)
Supplement: Supplementary file 1 [file foods-14-02165-s001.zip › List of chromatograms.pdf]

List of chromatograms

GC-MS_Fennel

GC-MS_Mentha

GC-MS_Thymus

HPLC_Fennel F1

HPLC_Fennel F3

HPLC_Mentha M2

HPLC_Mentha M4

HPLC_Thymus T2

HPLC_Thymus T4
