# Supplementary material for: Effect of Ascorbic Acid Addition on the Phenolic Compounds Content in Homogenates from Aerial Parts of Spearmint, Fennel, and Thyme
Source: Foods. 2025 Jun 21;14(13):2165. doi: 10.3390/foods14132165 (PMC12248822; doi:10.3390/foods14132165)
Supplement: Supplementary file 1 [file foods-14-02165-s001.zip › List of compounds in HPLC chromatograms.pdf]

List of compounds in HPLC chromatograms

Foeniculum vulgare

1 chlorogenic acid
2 miquelianin
3 quercetin derivative
4 1,5-dicaffeoylquinic acid
5 kaempferol-3-O-glucuronide
6 kaempferol-3-O-arabinoside

Mentha spicata

1 diosmin
2 hesperidin
3 rosmarinic acid
4 luteolin
5 apigenin
6 diosmetin

Thymus vulgare

1 luteolin-7-glucuronide
2 apigenin-7-glucuronide
3 rosmarinic acid
4 rosmarinic acid derivative 1
5 rosmarinic acid derivative 2
6 caffeoyl-rosmarinic acid
