# Supplementary material for: Effect of Ascorbic Acid Addition on the Phenolic Compounds Content in Homogenates from Aerial Parts of Spearmint, Fennel, and Thyme
Source: Foods. 2025 Jun 21;14(13):2165. doi: 10.3390/foods14132165 (PMC12248822; doi:10.3390/foods14132165)
Supplement: Supplementary file 1 [file foods-14-02165-s001.zip › List of the standards.pdf]

**Compound**	**Producer**	**Purity [%]**
t-Anethole	Sigma-Aldrich	99.5
p-Anisaldehyde	Sigma-Aldrich	98
Borneol	Sigma-Aldrich	99.0
Camphene	Supelco	?
Camphor	Sigma-Aldrich	99
3-Carene	Sigma-Aldrich	98.5
Carvacrol	Extrasynthese	98
Carvone	Sigma-Aldrich	98
t-Caryophyllene	Sigma-Aldrich	98.5
t-Caryophyllene oxide	Sigma-Aldrich	98.5
Chrysanthenol	Extrasynthese	?
p-Cymene	Sigma-Aldrich	99.5
Estragole	Extrasythese	97
Eucalyptol	Sigma-Aldrich	99
Ethylbenzene	Fluka	99
Fenchone	Extrasynthese	98
Geraniol	Sigma-Aldrich	98
α-Humulene	Sigma-Aldrich	95.0
Limonene	Sigma-Aldrich	97
Linalool	Extrasynthese	96.5
Menthol	Sigma-Aldrich	99
Menthone	Sigma-Aldrich	97.0
Myrcene	Sigma-Aldrich	90
Myrcenyl acetate	Sigma-Aldrich	95
α-Phellandrene	Sigma-Aldrich	95
α-Pinene	Sigma-Aldrich	98
ß-Pinene	Sigma-Aldrich	99.0
Sabinene hydrate	Sigma-Aldrich	97.0
Sabinyl acetate	Extrasynthese	90
γ-Terpinene	Extrasynthese	97
α+*ß*-Thujone	Sigma-Aldrich	α – 90-98, ß – 2-10
1-Tetradecene	Sigma-Aldrich	99.8

Terpinen-4-ol	Extrasynthese	95
α -Terpineol	Sigma-Aldrich	60.0-85.0
Terpinolene	Extrasynthese	95
Thymol	Extrasynthese	99
