# Supplementary figures and images for: Effect of Ascorbic Acid Addition on the Phenolic Compounds Content in Homogenates from Aerial Parts of Spearmint, Fennel, and Thyme
Source: Foods. 2025 Jun 21;14(13):2165. doi: 10.3390/foods14132165 (PMC12248822; doi:10.3390/foods14132165)

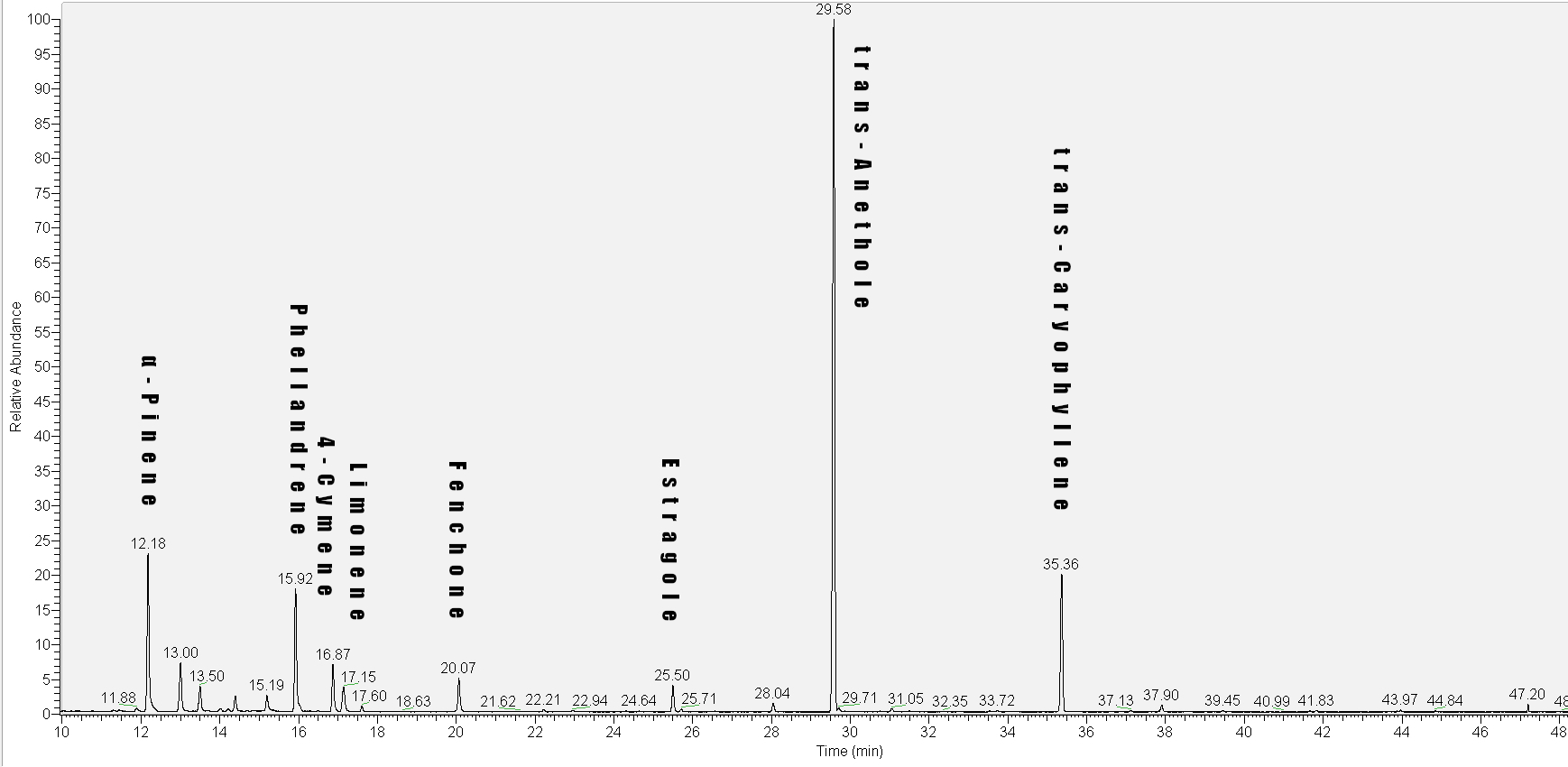

Supplement: Supplementary file 1 [file foods-14-02165-s001.zip › GC-MS_Fennel.jpg]

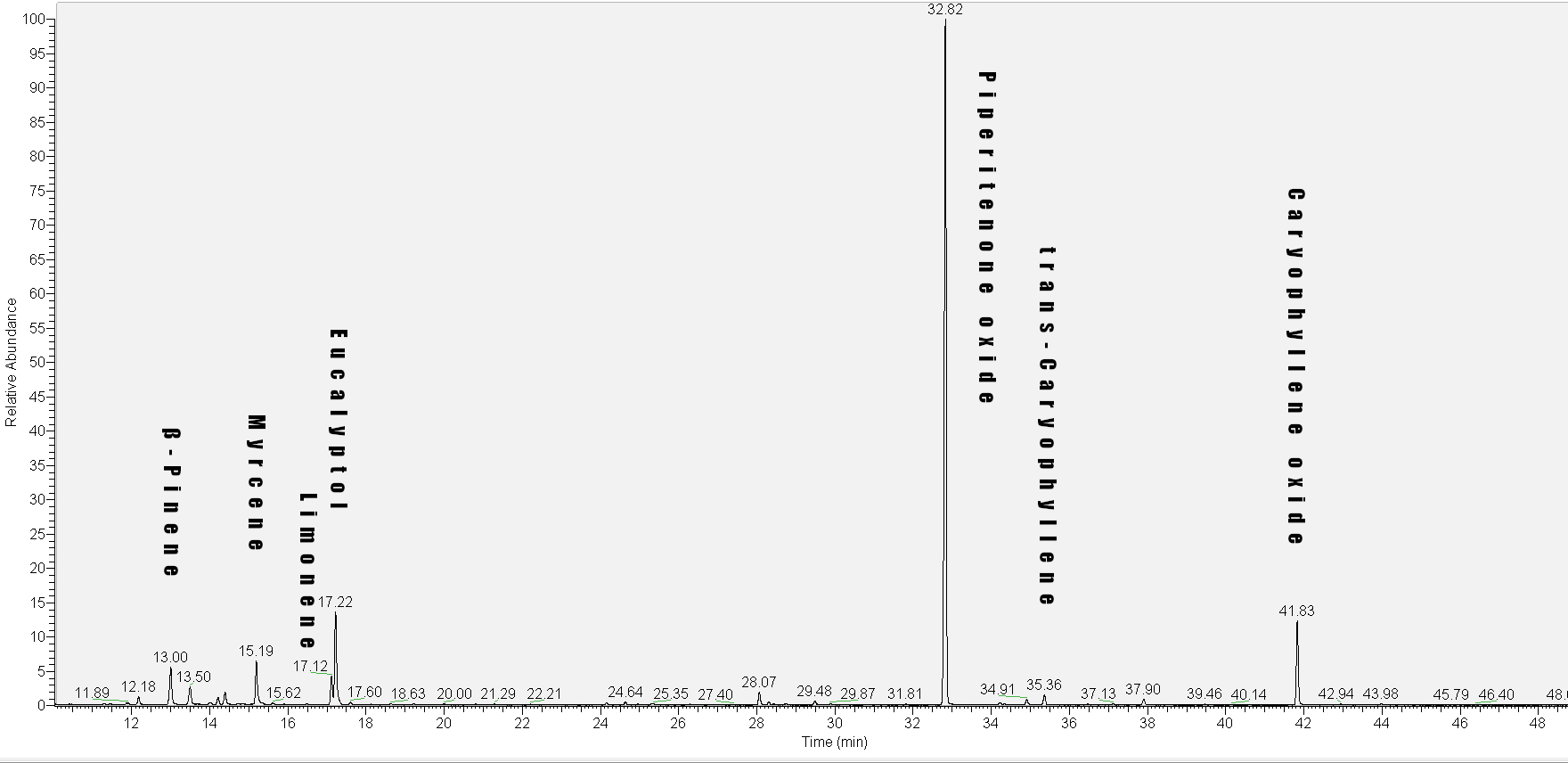

Supplement: Supplementary file 1 [file foods-14-02165-s001.zip › GC-MS_Mentha.jpg]

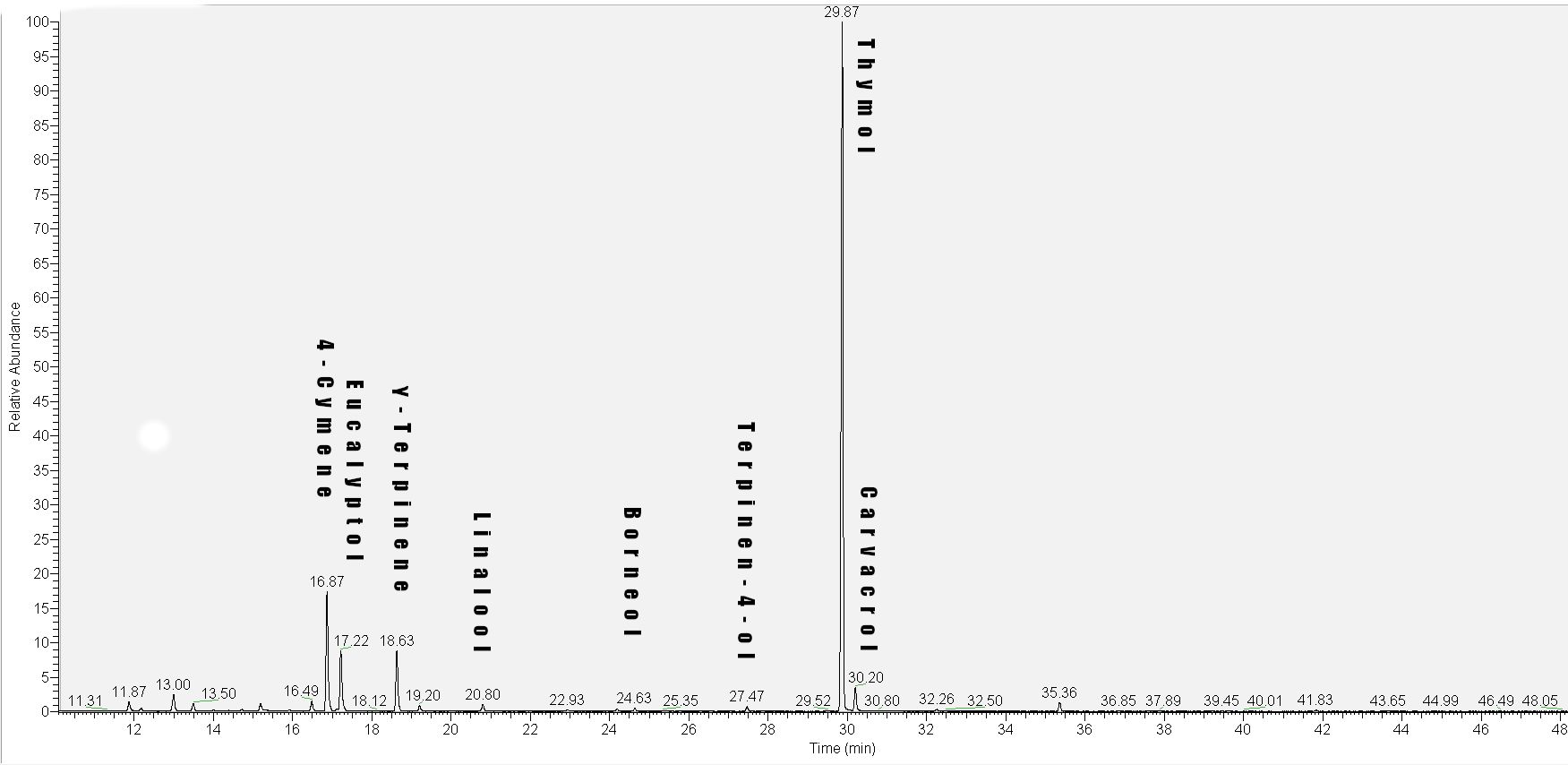

Supplement: Supplementary file 1 [file foods-14-02165-s001.zip › GC-MS_Thymus.jpg]

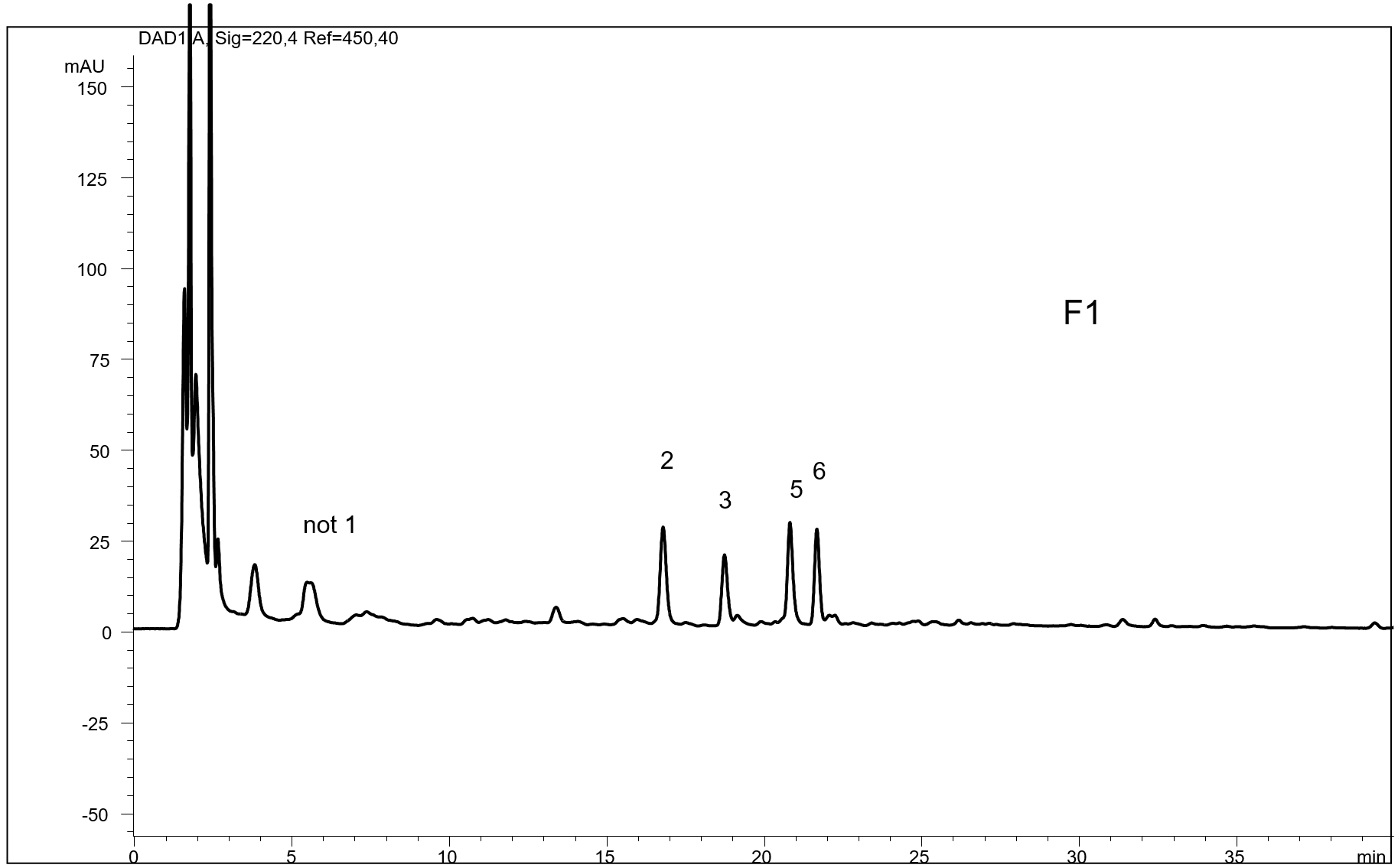

Supplement: Supplementary file 1 [file foods-14-02165-s001.zip › HPLC_Fennel F1.png]

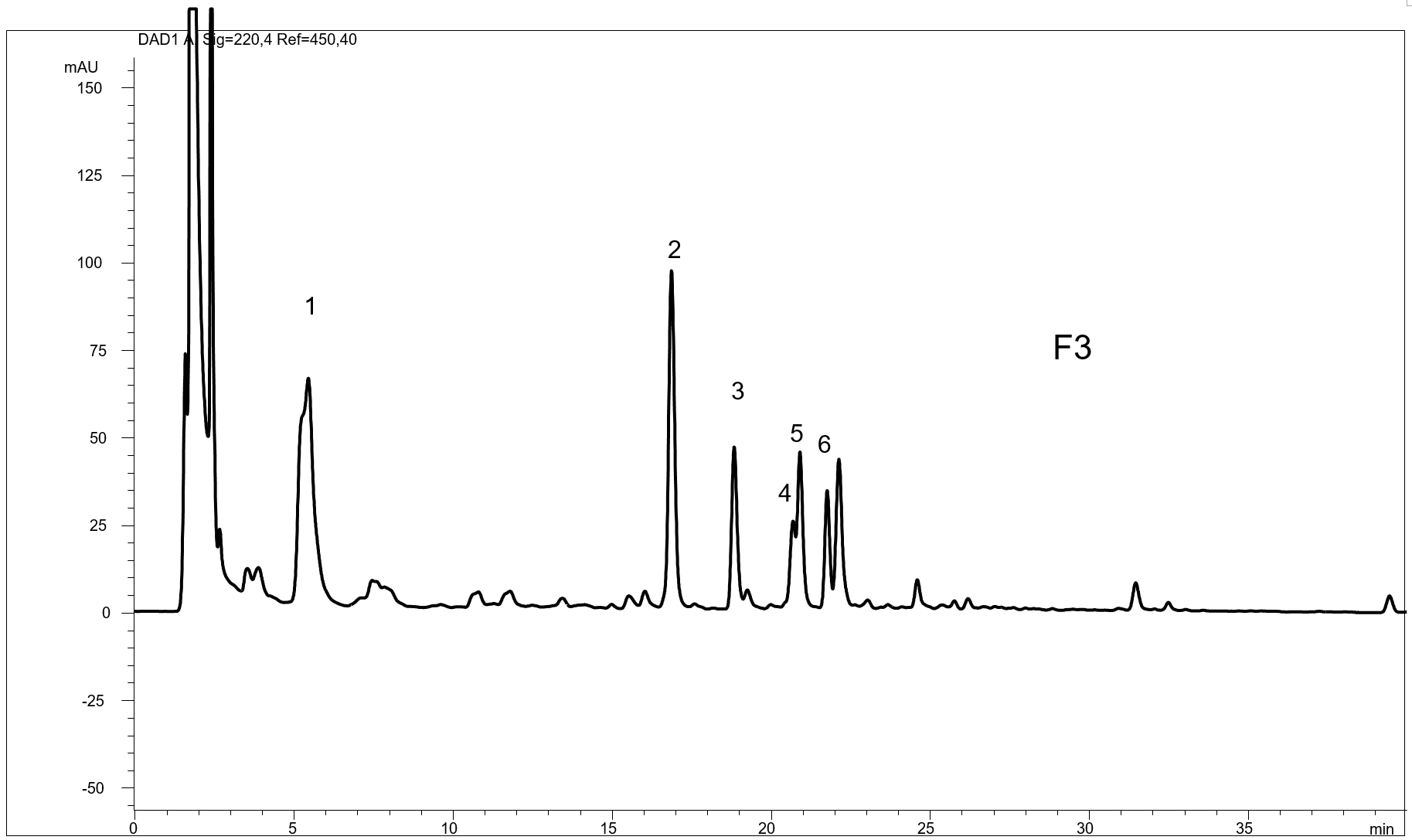

Supplement: Supplementary file 1 [file foods-14-02165-s001.zip › HPLC_Fennel F3.png]

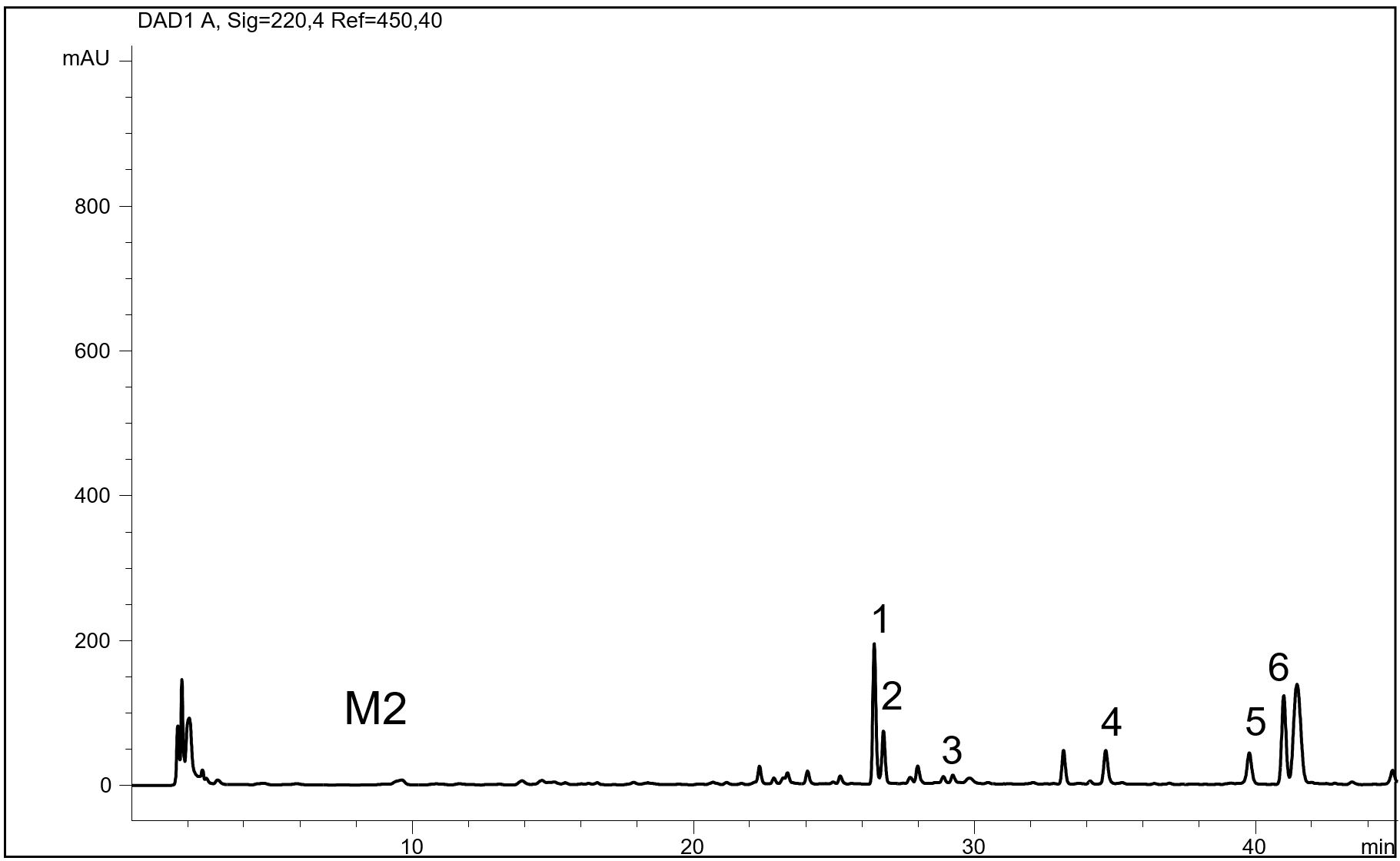

Supplement: Supplementary file 1 [file foods-14-02165-s001.zip › HPLC_Mentha M2.png]

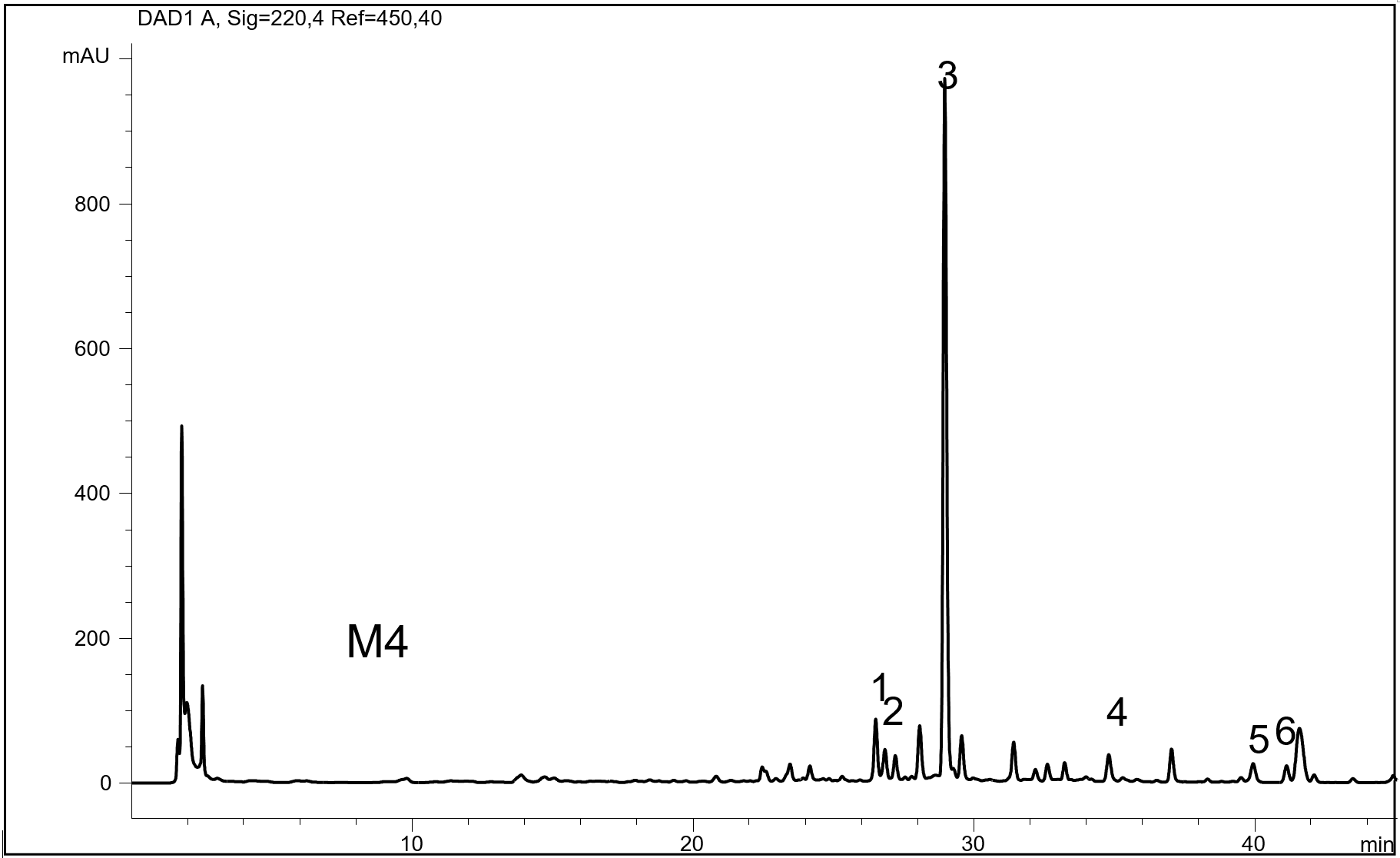

Supplement: Supplementary file 1 [file foods-14-02165-s001.zip › HPLC_Mentha M4.png]

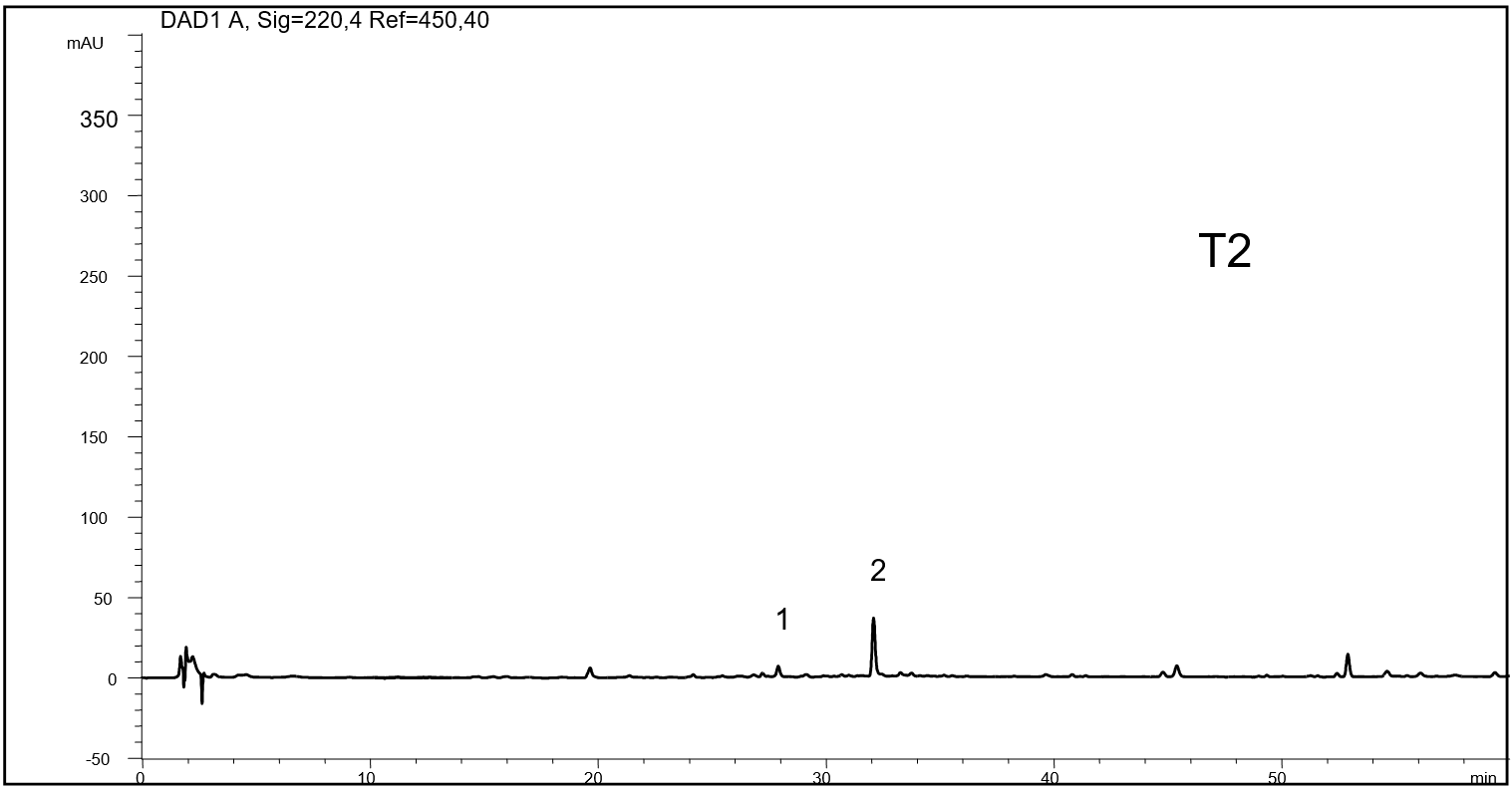

Supplement: Supplementary file 1 [file foods-14-02165-s001.zip › HPLC_Thymus T2.png]

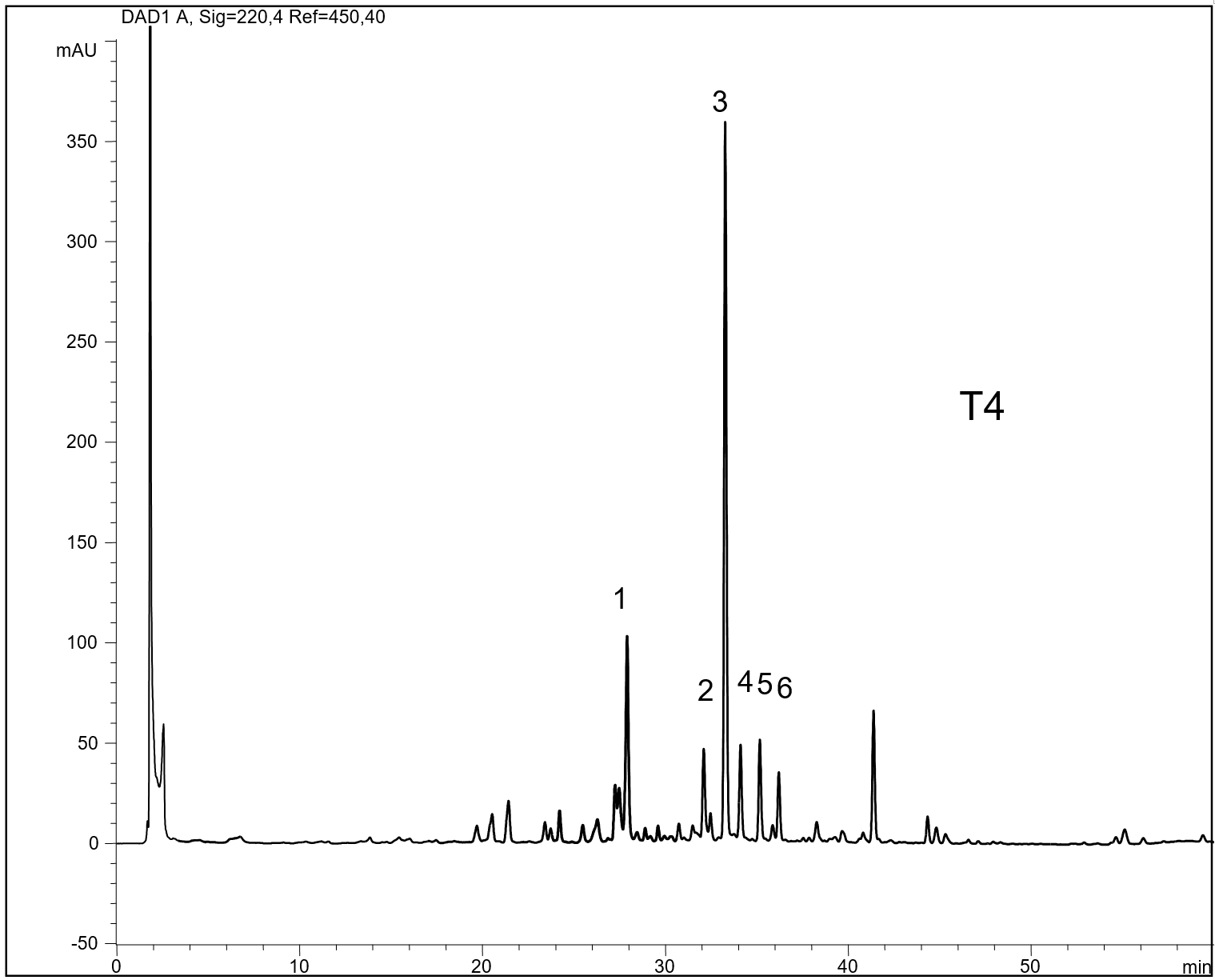

Supplement: Supplementary file 1 [file foods-14-02165-s001.zip › HPLC_Thymus T4.png]
